# Impact of Elevated Carbon Dioxide on Primary, Secondary Metabolites and Antioxidant Responses of *Eleais guineensis* Jacq. (Oil Palm) Seedlings

**DOI:** 10.3390/molecules17055195

**Published:** 2012-05-04

**Authors:** Mohd Hafiz Ibrahim, Hawa Z.E. Jaafar

**Affiliations:** Department of Crop Science, Faculty of Agriculture, University Putra Malaysia, 43400 Serdang, Selangor, Malaysia; Email: mhafizphd@yahoo.com

**Keywords:** oil palm seedlings, elevated CO_2_, primary and secondary metabolites, phenyl-alanine lyase (PAL), antioxidant

## Abstract

A split plot 3 by 3 experiment was designed to investigate the relationships among production of primary metabolites (soluble sugar and starch), secondary metabolites (total flavonoids, TF; total phenolics, TP), phenylalanine lyase (PAL) activity (EC 4.3.1.5), protein and antioxidant activity (FRAP) of three progenies of oil palm seedlings, namely *Deli AVROS*, *Deli Yangambi* and *Deli URT*, under three levels of CO_2_ enrichment (400, 800 and 1,200 µmol·mol^−1^) for 15 weeks of exposure. During the study, the treatment effects were solely contributed by CO_2_ enrichment levels; no progenies and interaction effects were observed. As CO_2_ levels increased from 400 to 1,200 µmol·mol^−1^, the production of carbohydrate increased steadily, especially for starch more than soluble sugar. The production of total flavonoids and phenolics contents, were the highest under 1,200 and lowest at 400 µmol·mol^−1^. It was found that PAL activity was peaked under 1,200 µmol·mol^−1^ followed by 800 µmol·mol^−1^ and 400 µmol·mol^−1^. However, soluble protein was highest under 400 µmol·mol^−1^ and lowest under 1,200 µmol·mol^−1^. The sucrose/starch ratio, *i.e.*, the indication of sucrose phosphate synthase actvity (EC 2.4.1.14) was found to be lowest as CO_2_ concentration increased from 400 > 800 > 1,200 µmol·mol^−1^. The antioxidant activity, as determined by the ferric reducing/antioxidant potential (FRAP) activity, increased with increasing CO_2_ levels, and was significantly lower than vitamin C and α-tocopherol but higher than butylated hydroxytoluene (BHT). Correlation analysis revealed that nitrogen has a significant negative correlation with carbohydrate, secondary metabolites and FRAP activity indicating up-regulation of production of carbohydrate, secondary metabolites and antioxidant activity of oil palm seedling under elevated CO_2_ was due to reduction in nitrogen content in oil palm seedling expose to high CO_2_ levels.

## 1. Introduction

*Elaeis guineensis* (Jacq.), known as oil palm, is the highest yielding vegetable oil crop in the World and is believed to originate from Africa [1]. Currently, Southeast Asia, particularly Malaysia and Indonesia, are the World’s largest producers of palm oil. In recent years, oil palm has gained wide recognition around the World because of its health implications and wide applications [2]. A recent study by Namvar *et al*. [3] has shown that oil palm leaves possess a strong estrogenic activity and cause a significant increase in percentage of vaginal cornification and uterine wet weight compared to control in a dose dependent manner in ovariectomized Sprague-Dawley rats. Furthermore, the study by Abeywardena *et al*. [4] demonstrated a promotion of vascular relaxation with consumption of oil palm leaf extract. The uptake of oil palm plant extract was also found to reduce the risk of breast cancer by inhibiting proliferation and growth of both MDA-MB-435 and MCF-7 cells in culture more effectively than α-tocopherol [5]. The health benefitd of oil palm are believed to be due to its flavonoids and phenolics content [6]. Flavonoids and phenolics are the most important groups of secondary metabolites and bioactive compounds in plants [7]. These compounds have been identified as natural antioxidants that may reduce oxidative damage to the human body [8]. Their function in human health is supported by the ability to induce human protective enzyme systems, and by a number of epidemiological studies suggesting protective effects against cardiovascular disease, cancers and other related diseases [9].

Several environmental factors such as nutrient supply, temperature, light conditions and atmospheric CO_2_ concentrations can influence the levels of total phenolics and flavonoids in plants [10]. As it is commonly known that secondary metabolites are associated with primary metabolism by the rates at which substrates are redirected from primary pathways to the secondary biosynthetic routes, several environmental factors affecting growth, photosynthesis and other parts of primary metabolism will also influence secondary metabolism [11]. One of these factors is CO_2_, and the increases in atmospheric CO_2_ due to climate change have a direct impact on plant secondary metabolites. Among such composition changes, most source-sink hypotheses (carbon nutrient balance hypothesis) [12] and growth-differentiation balance hypothesis [13] assume that elevated CO_2_ concentration supports a comparative increase in carbon accessibility that is accumulated in total non-structurable carbohydrate (TNC) and carbon based secondary metabolites (CBSM) when the provided carbon amounts exceed growth requirements [14]. Under optimal CO_2_ concentration conditions combined with nutrient resource restriction, which limit growths to a greater level than photosynthesis, plants showed an increase in the C/N ratios and excess of non-structural carbohydrates [15,16]. This excess may be available for the assimilation of CBSM.

High atmospheric CO_2_ concentrations often increase TNC concentrations in plants and possibly stimulate the secondary metabolism and antioxidant activity in plants [17]. Idso *et al*. [18], who evaluated the response of the tropical spider lily (*Hymenocallis littoralis*) to elevated levels of atmospheric CO_2_ over four growing seasons indicated that a 75% increase in the ambient CO_2_ concentration produced an 8% increase in pancratistatin, an 8% increase in *trans*-dihydronarciclasine, and a 28% increase in narciclasine that are efficient against lymphocytic leukemia and ovary sarcoma [19]. In the early studies of Barbale [20] and Madsen [21], a tripling of the atmospheric CO_2_ concentration produced a modest (7%) increase in antioxidants in the leaves and fruits of tomato plants. Hence, it can be concluded that exposure of plant to high levels of CO_2_ can increase the production of secondary metabolites and antioxidant.

Two hypotheses have been put forth to explain the increases of CBSM and antioxidants under high levels of CO_2_. Bass [22] considered that TNC in surplus of those required for protein production as the main factor affecting the increase of CBSM under elevated CO_2_. On the other hand, Jones and Hartley [23] proposed the contribution of the aminoacid phenylalanine as a common precursor for proteins and phenolics. The competition between proteins and phenolics for limiting phenylalanine would result in a swapping between protein and phenolics synthesis rates, thus, reversing the link between protein and phenolics distribution. These two hypotheses reflect on the increase of CBSM under elevated CO_2_ as a result of a metabolic excess of carbon with no physiological cost on growth. A negative relationship between CBSM and growth are anticipated because increases in growth along a gradient of increasing nutrient availability will result in less carbon being accessible for CBSM production [24].

There have been studies that investigated the effects of elevated CO_2_ on oil palm primary metabolism [25] but there have been no studies covering the response of oil palm secondary metabolites to increasing CO_2_. Although secondary metabolites constitute a significant sink for assimilated carbon, to date, there is unclear picture on how these compounds respond to different levels of elevated CO_2_. Previously, in Malaysia, the exposure of high than ambient CO_2_ have been found to enhance the secondary metabolites in Kacip Fatimah (*Labisia pumila*) [25,26,27,28] and ginger (*Zingiber officinale*) [29]. It would be expected that enrichment with elevated CO_2_ might enhance bioactive compounds in oil palm seedlings, thus, enhancing the medicinal values of this plant. With that in mind, the objective of this study was to examine the effects of different CO_2_ levels on the primary (soluble sugar, starch), secondary (total flavonoid and phenolics) metabolisms, and antioxidant activity (FRAP) of oil palm seedlings. The relationship between protein content, sucrose to starch ratio, phenyllalanine (PAL) activity, C/N ratio and antioxidative activity (FRAP) of oil palm seedling under elevated CO_2_ were also investigated, to understand the relationship between these parameters. 

## 2. Results and Discussion

### 2.1. Total Flavonoids and Phenolics Profiling

Carbon dioxide levels had a significant (*p* ≤ 0.05) impact on the production of total phenolics and flavonoids (Table 1) in oil palm seedlings. As CO_2_ levels increased from 400 to 1,200 µmol·mol^−1^ more total flavonoids and phenolics were produced. Oil palm had allocated more of the secondary metabolites to the leaves, followed by the stems and then the roots. In leaf, total flavonoids was enhanced by 86% and 132%, respectively, in 800 and 1,200 µmol·mol^−1^ compared to 400 µmol·mol^−1^ CO_2_. 

**Table 1:** Accumulation and partitioning of total flavonoids (TF) and total phenolics (TP) in different plant parts of *Elaeis guineensis* seedlings under different CO_2_ levels.

CO_2_ levels (µmol·mol^−1^)	Plant parts	Total flavonoid, TF (mg·g^−1^ rutin dry weight)	Total phenolics, TP (mg·g^−1^ gallic acid dry weight)
**400**	Leaf	0.10 ± 0.01 ^c^	3.81 ± 0.01 ^c^
	Stem	0.07 ± 0.02 ^d^	3.51 ± 0.02 ^c^
	Root	0.05 ± 0.03 ^d^	3.02 ± 0.01 ^c^
**800**	Leaf	0.19 ± 0.01 ^b^	5.86 ± 0.04 ^b^
	Stem	0.17 ± 0.02 ^b^	5.17 ± 0.04 ^b^
	Root	0.12 ± 0.02 ^c^	4.94 ± 0.05 ^b^
**1200**	Leaf	0.24 ± 0.02 ^a^	7.28 ± 0.07 ^a^
	Stem	0.23 ± 0.03 ^a^	6.87 ± 0.05 ^a^
	Root	0.20 ± 0.03 ^a^	6.55 ± 0.06 ^a^

All analyses are mean ± standard error of mean (SEM). N = 45. Means not sharing a common letter were significantly different at *p* ≤ 0.05. There were no progenies and interaction effects (CO_2_×Progenies) were observed during the study.

Total phenolics, on the other hand, increased by 52% to 91% under elevated CO_2_ compared to the ambient CO_2_ condition. This improvement in plant secondary metabolites might be due to increased total non structural carbohydrates (TNC) as exhibited by the correlation coefficient (R^2^ = 0.78; *p* ≤ 0.05) presented in Table 2, although a higher correlation coefficient (R^2^ = 0.98; *p* ≤ 0.05) was displayed by total soluble sugar than starch, implying that the accumulation of soluble sugar could be more responsible in the increase of plant secondary metabolites production than starch. 

**Table 2:** Correlation among the measured parameters in the study.

Parameters	1	2	3	4	5	6	7	8	9	10	11
**1.Flavonoid**	1.00										
**2. Phenolics**	0.87 *	1.00									
**3. Sucrose**	0.95 *	0.88 *	1.00								
**4. Starch**	0.88 *	0.76 *	0.67 **	1.00							
**5. TNC**	0.78 *	0.78 *	0.76 *	0.86 **	1.00						
**6. Suc/Starch**	−0.76 *	−0.75 *	0.67	0.68	0.57	1.00					
**7. PAL**	0.97 *	0.98 *	0.67	0.87 *	0.75 *	−0.72 *	1.00				
**8. Protein**	−0.87 *	−0.86 *	−0.79 *	−0.80 *	−0.75 *	0.84 *	−0.75 *	1.00			
**9. Nitrogen**	−0.77 *	−0.79 *	−0.84 *	−0.72 *	−0.77 *	0.84 *	−0.93 *	0.97 *	1.00		
**10. C/N**	0.89 *	0.89 *	0.76 *	0.77 *	0.81 *	0.12	0.86 *	−0.76 *	−0.87 *	1.00	
**11. FRAP**	0.98 *	0.98 *	0.77 *	0.76 *	0.75 *	0.14	0.89 *	−0.87 *	−0.88 *	0.78 *	1.00

Note TNC = total non structural carbohydrate; Suc/Starch = sucrose to starch ratio; PAL = phenyl alanine lyase activity; C/N = carbon to nitrogen ratio; FRAP = ferric reducing power; *, ** significant at *p* ≤ 0.05 and *p* ≤ 0.01 respectively.

This result was in conformity with Amin *et al.* [30] where they projected that the increase in secondary metabolites content in onion was due to 7% increase in total soluble sugar which was related to the enhanced polyphenolic production in onion by 21%. The increase in total flavonoids and phenolics compounds under elevated CO_2_ was also reported by Wang *et al*. [31] and Sttute *et al*. [32]. The current result suggest that enrichment with higher than ambient CO_2_ level is able to enhance the production of gallic acid and rutin in oil palm seedlings. This finding is important because these bioactive components in plants are important as they act as free radical scavengers, and hence can reduce the possibilities of major diseases such as cancers of leukemia, breast, bone and lung [33,34].

### 2.2. Total Soluble Sugar, Starch and Total Non Structural Carbohydrate Profiling

The accumulation and partitioning of carbohydrates were influenced by carbon dioxide levels(*p* ≤ 0.05). The accumulation of carbohydrates in different parts of the plant followed a descending order of leaf > stem > root. The concentration of total soluble sugar, starch and TNC increased with increasing CO_2_ enrichment levels (Table 3). Sucrose and starch contents registered the highest values in CO_2_-enriched palms at 800 µmol·mol^−1^ and 1,200 µmol·mol^−1^ CO_2_ compared to plants under raised under ambient conditions. Under high CO_2_ level (800 and 1,200 µmol·mol^−1^) conditions higher sucrose and starch were produced in the leaf, followed by the stem and then the root in comparison with the control treatment at 400 µmol·mol^−1^ CO_2_. Increase in the starch content in all parts of the oil palm seedlings was larger than the increase in sugar concentration [32]. The higher increase in the production of starch than soluble sugar under the exposure to elevated CO_2_ was also observed by other researchers [35,36,37,38,39] implying that starch production was more up-regulated under elevated CO_2_. 

**Table 3:** Accumulation and partitioning of soluble sugar, starch and total non structural carbohydrate (TNC) in different plant parts of *Elaeis guineensis* seedlings under different CO_2_ levels.

CO_2_ levels (µmol·mol^−1^)	Plant parts	Soluble sugar (mg·g^−1^ sucrose dry weight)	Starch (mg·g^−1^ glucose dry weight)	TNC (mg·g^−1^ dry weight)
**400**	Leaf	15.10 ± 0.55 ^c^	49.34 ± 0.98 ^c^	63.14 ± 1.31 ^d^
	Stem	11.32 ± 0.47 ^d^	40.24 ± 0.77 ^d^	51.57 ± 1.16 ^e^
	Root	9.34 ± 0.37 ^e^	34.34 ± 0.56 ^e^	12.68 ± 2.09 ^f^
**800**	Leaf	20.10 ± 0.16 ^b^	65.67 ± 0.57 ^b^	85.65 ± 0.99 ^b^
	Stem	18.76 ± 0.24 ^b^	59.67 ± 0.64 ^b^	76.78 ± 0.68 ^c^
	Root	16.32 ± 0.76 ^c^	57.16 ± 0.67 ^b^	72.67 ± 1.85 ^c^
**1200**	Leaf	27.97 ± 0.76 ^a^	77.24 ± 0.45 ^a^	103.21 ± 4.21 ^a^
	Stem	25.45 ± 0.36 ^a^	70.54 ± 0.35 ^a^	94.89 ± 4.12 ^a^
	Root	21.43 ± 0.13 ^b^	69.67 ± 0.32 ^a^	90.10 ± 4.32 ^a^

All analyses are mean ± standard error of mean (SEM). N = 45. Means not sharing a common letter were significantly different at *p* ≤ 0.05. There were no progenies and interaction effects (CO_2_×Progenies) were observed during the study.

The increased production of primary metabolites under elevated CO_2_ might be contributed by enhanced photosynthesis under high availability of CO_2_ that increases the availability of plant carbohydrates [28,29], however from the present study, soluble sugar, starch and TNC were observed to establish significant negative correlations with nitrogen with significant (*p* ≤ 0.05) respective values of R^2^ = −0.76, R^2^ = −0.81 and R^2^ = −0.76, which indicate that the exposure of oil palm to high CO_2_ level might dilute the nitrogen contents in the palm [25]. The accumulation of carbohydrate in CO_2_ enriched plant might be attributed to dilution of plant tissue nitrogen in enhanced plant growth under elevated CO_2_, especially, when nitrogen is limited. It is well known that nitrogen is important in regulation of the plant sink strength [24,25,35]. It is also well known that under elevated CO_2_ the nitrogen content would be reduced due to enhanced growth under this condition, that could reduce the sink size of the plant, hence, reducing the translocation of carbohydrates to other plant parts [40]. The reduction of nitrogen content might reflect either a higher nitrogen use efficiency due to reallocations of proteins, an ontogenetic drift leading to accelerated senescence as a results of faster growth, or inadequate nitrogen fertilization, uptake or assimilation [11]. An accumulation of total non-structural carbohydrates especially starch, C/N ratio and decrease in tissue nitrogen content is usually an indication of reduction of plant nitrogen content due to high growth rates under elevated CO_2_ [41,42,43,44]. The extra carbohydrates accumulated in oil palm seedlings in the present study might be channeled for the production of secondary metabolites (total phenolics and flavonoids). Carbohydrates are the basic compounds required to produce phenolics compound through shikimic acid pathway where extra carbohydrates derived from glycolysis and the penthose phosphate pathway are converted into the aromatic amino acid [45]. The up-regulation in carbon based secondary metabolites (CBSM) in the present study might be related to the balance between carbohydrate source and sink; as the greater the source-sink ratio is, the greater the production of secondary metabolites that might occur [46]. The current result suggested that under elevated CO_2_ the increase in production of TNC had improved the production of CBSM in oil palm seedlings.

### 2.3. Sucrose/Starch Ratio

The sucrose-to-starch ratio was only substantially (*p* ≤ 0.05) influenced by CO_2_ levels. The sucrose/starch ratio in 400 µmol·mol^−1^ CO_2_ was 14% and 21% lower in 800 and 1,200 µmol·mol^−1^, respectively (Table 4).

**Table 4:** Effects of different CO_2_ concentrations on some biochemical parameters in *Elaeis guineensis* seedlings.

Parameters	400 µmol·mol^−1^	800 µmol·mol^−1^	1200 µmol·mol^−1^
Sucrose/Starch ratio	0.390 ± 0.031 ^a^	0.334 ± 0.244 ^b^	0.308 ±0.343 ^c^
PAL activity (nM transcinnamic mg^−1^ protein h^−1^)	17.76 ± 0.02 ^c^	22.89 ± 0.12 ^b^	30.93 ± 0.07 ^a^
Protein content (mg/g fresh weight)	12.27 ± 1.22 ^a^	8.87 ± 0.87 ^b^	3.17 ± 0.21 ^c^
Leaf nitrogen (%)	2.96 ± 0.17 ^a^	1.63 ± 0.06 ^b^	0.97 ± 0.01 ^c^
C/N ratio	9.33 ± 0.77 ^c^	19.07 ± 0.98 ^b^	34.55 ± 2.34 ^a^

All analyses are mean ± standard error of mean (SEM), N = 45. Means not sharing a common single letter were significantly different at *p* ≤ 0.05. There were no progenies and interaction effects (CO_2_×Progenies) were observed during the study.

These results illustrated that as the CO_2_ levels increased to reach 1,200 µmol·mol^−1^ the sucrose/starch ratio conversely decreased. Sucrose/starch ratio is an indication of sucrose phosphate synthase activity (SPS) in plants [47]. In the present study, the sucrose/starch ratio *i.e*., the indication of SPS activity was shown to be lowest in 1,200 µmol·mol^−1^ CO_2_ and highest in 400 µmol·mol^−1^CO_2_. As indicated in the present work, SPS activity was the lowest when the level of CO_2_ fertilization was the highest. At the same time, it was also noted that sucrose/starch ratio had a significant (*p* ≤ 0.05) positive relationship with nitrogen and protein (R^2^ = 0.84). These results signify that SPS activity was higher in plants that contained high nitrogen and protein contents. A similar result as in the present study was also observed by Cruz *et al*. [48], which imply that the down regulation of the SPS activity in oil palm seedlings could occur under high level of CO_2_ enrichment that might due to reduction in nitrogen levels under elevated CO_2_ levels [11].

### 2.4. Phenylalanine Lyase (PAL) Activity

The PAL activity was largely influenced by CO_2_ levels (*p* ≤ 0.05). The highest PAL activity was found to be at 1,200 µmol·mol^−1^ (30.93 nM *trans*-cinnamic mg^−1^ protein h^−1^), followed by 800 µmol·mol^−1^ (22.89 nM *trans*-cinnamic mg^−1^ protein h^−1^) and the lowest at 400 µmol·mol^−1^ with only 17.76 nM *trans*-cinnamic mg^−1^ protein h^−1^ (Table 4). PAL had also established a considerable (*p* ≤ 0.05) positive relationship with total phenolics (R^2^ = 0.744) and flavonoids (R^2^ = 0.842), which might indicate an up-regulation of plant secondary metabolite production with increased PAL activity. This is basically true by the fact that phenylalanine is a precursor of total phenolics and flavonoids biosynthesis [26]. These results suggested that the up-regulation of the plant secondary metabolites production in oil palm seedlings under high CO_2_ could be due to an increase in the PAL activity. The increase in PAL activity in the current study might also be related to reduction in nitrogen content in plant exposed to high CO_2_ levels which could have increased the availability of phenylalanine (phe) due to restriction in the protein production that enhanced phenylalanine partitioning to the production of CBSM [49]. This statement is supported by the significant negative correlation between PAL and nitrogen (R^2^ = −0.93; *p* ≤ 0.05) as shown in the correlation Table 2. These results suggested that the up-regulation of the production of plant secondary metabolites in oil palm seedlings under high CO_2_ levels might be due to the increase in PAL activity that was triggered by the reduced nitrogen content when seedlings were exposed to higher than ambient CO_2_ level. 

### 2.5. Soluble Protein

In contrast to PAL, soluble protein of oil palm seedling was inhibited by the CO_2_ levels (*p* ≤ 0.05). As the levels of CO_2_ increased from 400 to 1,200 µmol·mol^−1^ the soluble protein decreased. The highest protein content was obtained at 400 µmol·mol^−1^ CO_2_ (12.27 mg·g^−1^ dry weight) and the lowest was at 1,200 µmol·mol^−1^ CO_2_ (3.17 mg·g^−1^ dry weight; Table 4). Protein content was also observed to have a negative relationship with total phenolics and flavonoids (R^2^ = −0.87; R^2^ = −0.76, respectively; *p* ≤ 0.05; Table 2) indicating the possible occurrence of up-regulation of plant secondary metabolites under reduced protein content [47]. Decrease in protein production under high CO_2_ and low nitrogen levels as exhibited by the present work, might decrease the usage of PAL in protein synthesis, hence, channeling it for the biosynthesis of plant secondary metabolites [27]. A similar result to the one presented here was also observed by Robredo *et al*. [50] in barley, where the highest protein accumulation was found under ambient CO_2_; thus suggesting that a restriction in protein synthesis under elevated CO_2_ could enhance the production of total phenolics and flavonoids in oil palm seedlings.

### 2.6. Leaf Nitrogen and C/N Ratio

The increase in CO_2_ levels had significantly lowered the nitrogen levels while improving the C/N ratio of oil palm seedlings (*p* ≤ 0.05). There were no progenies and interaction effects observed in this work. The nitrogen levels in the leaves of oil palm seedlings exposed to high CO_2_ levels registered lower values of 1.63% and 0.97% in the 800 and 1,200 µmol·mol^−1^ treatments, respectively, compared to 400 µmol·mol^−1^ CO_2_ condition that recorded 2.97%. As the CO_2_ enrichment levels increased from 400 to 1,200 µmol·mol^−1^ leaf C/N also increased considerably (Table 4). The increase in C/N under high CO_2_ usually contributed more to reduction in nitrogen content than increase in carbon content [51]. Carbon to nitrogen ratio in 400 µmol·mol^−1^ CO_2_ was 104% and 270% lower than those in 800 and 1,200 µmol·mol^−1^ CO_2_, respectively. A similar increase in C/N ratio of plants enriched with high CO_2_ was also observed by Fonseca *et al*. [38] in *Plantago major*. 

Carbon to nitrogen ratio had a significant positive relationship (*p* ≤ 0.05) with total flavonoids and phenolics compounds (R^2^ = 0.91; Table 2), signifying a good direct association between the C/N ratio and plant secondary metabolites. In the present study, C/N ratio was shown to have a significant positive correlation with PAL activity (R^2^ = 0.86; *p* ≤ 0.05) implying that when the C/N ratio increased, the production of secondary metabolites might also be expected to increase due to high PAL activity [27]. Winger *et al.* [52] indicated that increases in the C/N ratio in plants were an indication of increases in the synthesis of plant secondary metabolites, especially phenolics and flavonoids. The increase in plant C/N ratio signifies that an increase in the production of TNC could have stimulated the production of plant secondary metabolites [25]. 

### 2.7. Reducing Ability Ferric Reducing Antioxidant Potential (FRAP)

Several methods are known to measure the total antioxidant capacity of plants, including the ferric reducing/antioxidant potential (FRAP) assay, which has been adopted in this study. The FRAP assay depends upon the reduction of ferric tripyridyltriazine [Fe(III)-TPTZ] complex to the ferrous tripyridyltriazine [Fe(II)-TPTZ] by a reductant at low pH. The FRAP assay has been used widely to estimate the antioxidant component or power in dietary polyphenols [53]. The FRAP activities in oil palm seedlings was influenced by CO_2_ levels (*p* ≤ 0.01). The reducing power for the different parts (leaves, stems and roots) of oil palm extracts was in the range of 532.21 to 777.43 μM of Fe (II)/g^−1^ dry weight (Table 5).

**Table 5:** Total antioxidant (FRAP) activity in different parts of three varieties of *Elaeis guineensis* under different CO_2_ levels. BHT, α-tocopherol and vitamin c were used as positive controls.

CO_2_ levels (µmol·mol^−1^)	Extract source	FRAP activity (µM Fe (II)/g dry weight)
	Leaf	582.42 ± 1.65 ^e^
**400**	Stem	558.14 ± 1.09 ^e^
	Root	532.21 ± 1.08 ^e^
	Leaf	688.83 ± 1.05 ^d^
**800**	Stem	675.11 ± 0.98 ^d^
	Root	647.73 ± 0.43 ^d^
	Leaf	777.43 ± 0.23 ^c^
**1200**	Stem	744.74 ± 0.98 ^c^
	Root	704.31 ± 1.21 ^c^
	BHT	88.81 ± 10.34 ^f^
**Controls**	α-tocopherol	998.41 ± 41.24 ^b^
	Vitamin C	2879.67 ± 56.78 ^a^

All analyses are mean ± standard error of mean (SEM), N = 45. Means not sharing a common single letter were significantly different at *p* ≤ 0.05.

Increasing CO_2_ concentration in the growth climate had significant effect on FRAP activities and the profiling in different palm parts. As the levels of CO_2_ increased, the FRAP activities were improved under elevated CO_2_ treatments (800 and 1,200 µmol·mol^−1^) that registered 777.43 to 647.73 μM of Fe (II)/g^−1^ dry weight, Meanwhile, the activities of FRAP in 400 µmol·mol^−1^ just ranged from 532.21 to 582.42 μM of Fe (II)/g^−1^ dry weight, suggesting that the elevated CO_2_ conditions were able to enhance the antioxidative properties of the oil palm seedlings. However, the FRAP values for the leaf, stem and root extracts in three levels of CO_2_ concentrations (400, 800 and 1,200 µmol·mol^−1^) were significantly lower than those of the controls viz. vitamin C [2879.67 μM Fe (II)/g−1] and α-tocopherol [998.41 μM Fe (II)/g−1], but higher than that of BHT [88.81 μM Fe (II)/g−1]. In all CO_2_ concentrations, the FRAP activity was found to be highest in the leaf followed by stem and the root. The increase in the foliar activity might be due to high total phenolics and flavonoids content in this plant part [47]. This finding was justified by significant positive correlations between FRAP and total phenolics and total flavonoids (R^2^ = 0.98; *p* ≤ 0.01), implying that the enhanced antioxidant activity of oil palm seedlings under high levels of CO_2_ enrichment could be related to the plants increased hydrogen donating abilities [54,55,56,57]. In a previous study in mengkudu, a strong positive relationship between total phenolics contents and antioxidant activity, which appear to be the trend in many plant species, was reported by Mohd Zin *et al*. [57]. 

The result from the present study was in agreement with Wang *et al*. [31] who reported that the free radical scavenging power of strawberry increased at elevated CO_2_ concentration. The current study had shown that enrichment with high CO_2_ levels could enhance the antioxidant activities of oil palm seedlings, particularly in the leaf, which could be used as a radical inhibitor or scavenger, acting possibly as a primary antioxidant [26]. The correlations in Table 2 have shown that nitrogen had a significant negative relationship with carbohydrate, secondary metabolites and FRAP activity. This implies that the enhancement of syntheses of carbohydrates, secondary metabolites and antioxidant activity of oil palm seedlings under higher than ambient CO_2_ was possibly due to declining in nitrogen content in plant tissue when seedlings exposed to elevated CO_2_ [25]. The reduction in nitrogen content of plants under higher than ambient CO_2_ could have been caused by the accelerated growth that diluted plant nitrogen content [24]. In the present study, this condition was shown to have enhanced the primary, secondary and antioxidant activities in the oil palm seedlings, hence, possibly enhancing the medicinal properties of this plant.

## 3. Experimental

### 3.1. Experimental Location, Plant Materials and Treatments

The experiments were carried out at the Malaysian Palm Oil Board (MPOB) Headquarters in Bandar Baru Bangi Selangor, Malaysia. The site is situated at longtitude 101°44'N and latitude 2°58'S, 68 m above sea level with a mean atmospheric pressure of 1.013 kPa, mean daily temperature of 30 °C, mean relative humidity of 70% and maximum and minimum daytime irradiance of 1,600 and 40 µmol·m^−2^·s^−1^, respectively. Three month-old Tenera (*Dura x Pesifera*) oil palm progenies of *Deli Urt*, *Deli Yangambi* and *Deli AVROS* were left for two months to acclimatize in a nursery until ready for the treatments. Carbon dioxide enrichment treatments started onto five month-old seedlings by exposing them to three levels of CO_2_, viz. ambient CO_2_ at 400 µmol·mol^−1^, twice ambient CO_2_ at 800 µmol·mol^−1^ and thrice ambient CO_2_ at 1,200 µmol·mol^−1^. The split plot 3 × 3 factorial experiment was designed using randomized complete block design with CO_2_ levels being the main plot and progenies as the sub-plot replicated three times. Each experimental unit (3 × 3 × 3 = 27) were represented with five plants totaling 135 plants were used during the experiment. Carbon dioxide at 99.8% purity was supplied from a high-pressure CO_2_ cylinder and injected through a pressure regulator into fully sealed 2 m × 3 m growth compartment at 2 d^−1^ continuously from 08:00 to 10:00 [54]. During CO_2_ exposition period, the CO_2_ concentration at different enrichment treatments was measured using VaisalaTM (Ouijart, Finland) CO_2_ portable meter.

### 3.2. Total Phenolics and Total Flavonoids Quantification

The method of extraction and quantification for total phenolics and flavonoids contents followed after Ibrahim *et al.* [58]. An amount of ground tissue samples (0.1 g) was extracted with 80% ethanol (10 mL) on an orbital shaker for 120 min at 50 °C. The mixture was subsequently filtered (Whatman™ No.1), and the filtrate was used for the quantification of total phenolics and total flavonoids. Folin-Ciocalteu reagent (diluted 10-fold) was used to determine the total phenolics content of the leaf samples. Two hundred µL of the sample extract was mixed with Follin-Ciocalteau reagent (1.5 mL) and allowed to stand at 22 °C for 5 min before adding NaNO_3_ solution (1.5 mL, 60 g·L^−1^). After two hours at 22 °C, absorbance was measured at 725 nm. The results were expressed as mg·g^−1^ gallic acid equivalent (mg GAE g^−1^ dry sample). For total flavonoids determination, a sample (1 mL) was mixed with NaNO_3_ (0.3 mL) in a test tube covered with aluminum foil, and left for 5 min. Then 10% AlCl_3_ (0.3 mL) was added followed by addition of 1 M NaOH (2 mL). Later, the absorbance was measured at 510 nm using a spectrophometometer with rutin as a standard (results expressed as mg·g^−1^ rutin dry sample).

### 3.3. Soluble Sugar Determination

Soluble sugar were measured spectrophotometrically using the method described by Ibrahim *et al.* [59]. Samples (0.5 g) were placed in 15 mL conical tubes. Then distilled water (10 mL) was added and the mixture was then vortexed and incubated for 10 min. Anthrone reagent was prepared using anthrone (0.1 g) that was dissolved in 95% sulphuric acid (50 mL). Sucrose was used as a standard stock solution to prepare a standard curve for the quantification of sucrose in the sample. The mixed sample of ground dry sample and distilled water was centrifuged at a speed of 3,400 rpm for 10 m and then filtered to get the supernatant. To an aliquot (4 mL) of the sample, anthrone reagent (8 mL) was added and the mixture was placed in a water bath set at 100 °C for 5 min before the sample was measured at absorbance 620 nm using UV160U spectrophotometer (Shimadzu, Kyoto, Japan). The soluble sugar in the sample was expresses as mg sucrose g^−1^ dry sample.

### 3.4. Starch Determination

Starch content was determined spectrophometrically using method by Thayumanavam and Sadasivam [60]. Dry sample (about 0.5 g) was homogenized in hot 10 mL 80% ethanol to remove the sugar. The sample was then centrifuged at 5,000 rpm for 5 min and then the residue was retained. After that, distilled water (5.0 mL) and 52% perchloric acid (6.5 mL) were added to the residue, then the solution was centrifuged and the supernatant separated and then filtered through No. 5 filter paper (Whatman, Chicago, IL, USA). The processes were repeated until the supernatant was made up to 100 mL. An aliquot of the supernatant (100 µL) was added to distilled water until the volume became 1 mL. After that, 4 mL anthrone reagent (Sigma, Columbus, OH, USA; prepared with 95% sulphuric acid by adding 2 g of anthrone to 100 mL 95% sulphuric acid) was added to a tube. The mixed solution was placed in the water bath at 100 °C for eight min and then cooled to temperature room before sample read at absorbance of 630 nm to determine the sample starch content. Glucose was used as a standard and starch content was expressed as mg glucose equivalent g^−1^ dry sample. 

### 3.5. Total Non Structural Carbohydrate (TNC) and Sucrose to Starch Ratio

The total non structural carbohydrate was calculated as the sum of total soluble sugar and starch content. The sucrose to starch ratio was calculated by dividing sucrose to starch content [61].

### 3.6. Phenylalanine-Ammonia-Lyase (PAL) Activity

Phenylalanine-ammonia-lyase (PAL) activity was measured using the method described by Ibrahim and Jaafar [27]. The enzyme activity was determined by measuring spectrophotometrically the production of *trans*-cinnamic acid from L-phenylalanine. Enzyme extract (10 µL) was incubated at 40 °C with 12.1 mM L-phenylalanine (90 µL, Sigma) that were prepared in 50 mM Tris-HCl, (pH 8.5). After 15 min of reaction, *trans*-cinnamic acid yield was estimated by measuring increase in the absorbance at 290 nm. Standard curve was prepared by using a *trans*-cinnamic acid standard (Sigma) and the PAL activity was expressed as nM *trans*-cinnamic acid µg^−1^ protein h^−1^.

### 3.7. Soluble Protein Determination

Protein content was determined using the method of Bradford [62]. In this method, fresh leaf samples (about 2 g) were cut into pieces using scissors and grounded in mortar with 0.05 M Tris buffer (1 mL, pH 8.5) and powdered with liquid nitrogen. The homogenate was then centrifuged at 9,000 rpm for 10 min and then stored under refrigeration at 4 °C for 24 h. After the extraction, supernatant from the samples (about 100 µL) was added to Bradford reagent (3 mL, Sigma, prepared using 10 mL of the reagent diluted with 50 mL distilled water) and then incubated for 5 min before being measured at 595 nm with the spectrophotometer. In this method bovine serum (Sigma) was used as a standard to produce calibration curve between actual protein content and spectrophotometer readings. The protein was expressed as mg g^−1^ protein fresh weight.

### 3.8. Total Carbon, Nitrogen and C:N Ratio

Total carbon and C:N ratio were measured by using a CNS 2000 analyzer (Model A Analyst 300, LECO Inc, Houston, TX, USA). This was performed by placing ground leaf sample (0.05 g) into the combustion boat. Successively, the combustion boat was transferred to the loader before the sample was burned at 1,350 °C to obtain the reading of total carbon and nitrogen content of the samples [63].

### 3.9. Reducing Ability (FRAP Assay)

The ability to reduce ferric ions was measured using modifying methods of Benzie and Strain [64]. An aliquot (200 µL) of the extract with appropriate dilution was added to FRAP reagent (3 mL, 10 parts of 300 mM sodium acetate buffer at pH 3.6, 1 part of 10 mM TPTZ solution and 1 part of 20 mM FeCl_3_·6H_2_O solution) and the reaction mixture was incubated in a water bath at 37 °C. The increase in absorbance at 593 nm was measured after 30 min. The antioxidant capacity based on the ability to reduce ferric ions of the extract was expressed as in µM Fe (II)/g^−1^ dry mass and compared with those of standards for BHT, ascorbic acid, and α-tocopherol.

### 3.10. Statistical Analysis

Data were analyzed using analysis of variance by SAS version 17. Mean separation test between treatments was performed using Duncan multiple range test and standard error of differences between means was calculated with the assumption that data were normally distributed and equally replicated [65,66,67].

## 4. Conclusions

The current work has demonstrated that high levels of CO_2_ are able to alter the synthesis of flavonoids and phenolics in oil palm seedlings. As CO_2_ levels increased from 400 to 1,200 µmol·mol^−1^ PAL activity was enhanced, which had substantially improved the production of total flavonoids and phenolics. The increase in PAL activity for secondary metabolites synthesis under high CO_2_ might be due to restriction in the production of protein as nitrogen content was reduced under speedy growth, hence, increasing the availability of phenylanine for phenolics and flavonoids syntheses. The increase in production of secondary metabolites under high CO_2_ was manifested with high C/N ratio and high antioxidant activity in oil palm seedlings that increases the medicinal value of this plant. 
